# Sugar alcohol provides imaging contrast in cancer detection

**DOI:** 10.1038/s41598-019-47275-5

**Published:** 2019-07-31

**Authors:** Puneet Bagga, Neil Wilson, Laurie Rich, Francesco M. Marincola, Mitchell D. Schnall, Hari Hariharan, Mohammad Haris, Ravinder Reddy

**Affiliations:** 1 Center for Magnetic Resonance and Optical Imaging, Department of Radiology, University of Pennsylvania, Philadelphia, PA USA; 2 Research Branch, Sidra Medical and Research Center, Doha, Qatar; 3 Laboratory Animal Research Center, Qatar University, Doha, Qatar

**Keywords:** Translational research, Cancer imaging

## Abstract

Clinical imaging is widely used to detect, characterize and stage cancers in addition to monitoring the therapeutic progress. Magnetic resonance imaging (MRI) aided by contrast agents utilizes the differential relaxivity property of water to distinguish between tumorous and normal tissue. Here, we describe an MRI contrast method for the detection of cancer using a sugar alcohol, maltitol, a common low caloric sugar substitute that exploits the **c**hemical **e**xchange **s**aturation **t**ransfer (CEST) property of the labile hydroxyl group protons on maltitol (malCEST). *In vitro* studies pointed toward concentration and pH-dependent CEST effect peaking at 1 ppm downfield to the water resonance. Studies with control rats showed that intravenously injected maltitol does not cross the intact blood-brain barrier (BBB). In glioma carrying rats, administration of maltitol resulted in the elevation of CEST contrast in the tumor region only owing to permeable BBB. These preliminary results show that this method may lead to the development of maltitol and other sugar alcohol derivatives as MRI contrast agents for a variety of preclinical imaging applications.

## Introduction

Medical imaging is widely used to monitor structural, functional, and molecular changes in cancer and the use of contrast agents has significantly improved the detection by providing enhanced contrast between normal and pathological tissues^1–4^. Positron Emission Tomography using ^18^Fluoro-2-deoxy-glucose (^18^FDG-PET) combined with either computed tomography (CT) or magnetic resonance imaging (MRI) has gained widespread application as a molecular and metabolic imaging modality of cancers based on the high glycolytic activity of tumors^5,6^. However, owing to the high metabolic activity of surrounding neurons, ^18^F-FDG uptake in the normal brain tissue limits its use for the imaging of cerebral gliomas^7^. In addition to conventional MRI, dynamic contrast enhanced (DCE) MRI utilizes the relaxivity perturbation potential of gadolinium-based contrast agents (GBCAs) to detect and characterize cancer^8^. Although, recent studies have reported the deposition of GBCAs in the brain and bone matrix^3,9,10^, further studies are required to evaluate the long-term effects of gadolinium (Gd) accumulation on normal tissue function.

The Chemical Exchange Saturation Transfer (CEST) MRI technique probes the exchange of labile protons of the solute with bulk water protons^11–15^. By applying low-power frequency-selective radio-frequency (RF) pulses for a long time, magnetization of exchangeable protons on a metabolite can be saturated. The chemical exchange mediated accumulation of these saturated protons with water decreases the bulk water signal in a concentration and pH dependent manner^14,16,17^. The difference in the water signal obtained with and without RF saturation can be measured as the CEST contrast^14,15^. CEST MRI has been used to image different metabolites and macromolecules *in vivo*, with applications in several human disorders^18–23^. Since the CEST method provides orders of magnitude higher sensitivity than traditional proton MR spectroscopy (^1^H MRS), it enables detection of subtle changes in the level of metabolite of interest^14,17^. Various groups have reported the use of glucose and its analogues as CEST contrast agent to study cancer and neurodegeneration^18,20,24–34^.

In this study, we introduce a new contrast agent, maltitol, a sugar alcohol commonly used as a sweetener due to its less caloric value. We exploited the CEST behavior of labile hydroxyl (-OH) protons on maltitol with those of the bulk water and termed this new method as malCEST. The concentration and pH dependence of malCEST contrast was measured *in vitro* in solution phantoms. The potential of malCEST as an MR imaging method to image cancer was assessed in a rat glioma model and compared with gadolinium-diethylenetriamine-pentaacetic-acid (Gd-DTPA) contrast enhanced MRI.

## Results

### Chemical evaluation of exchangeable protons of maltitol

**Figure 1:**
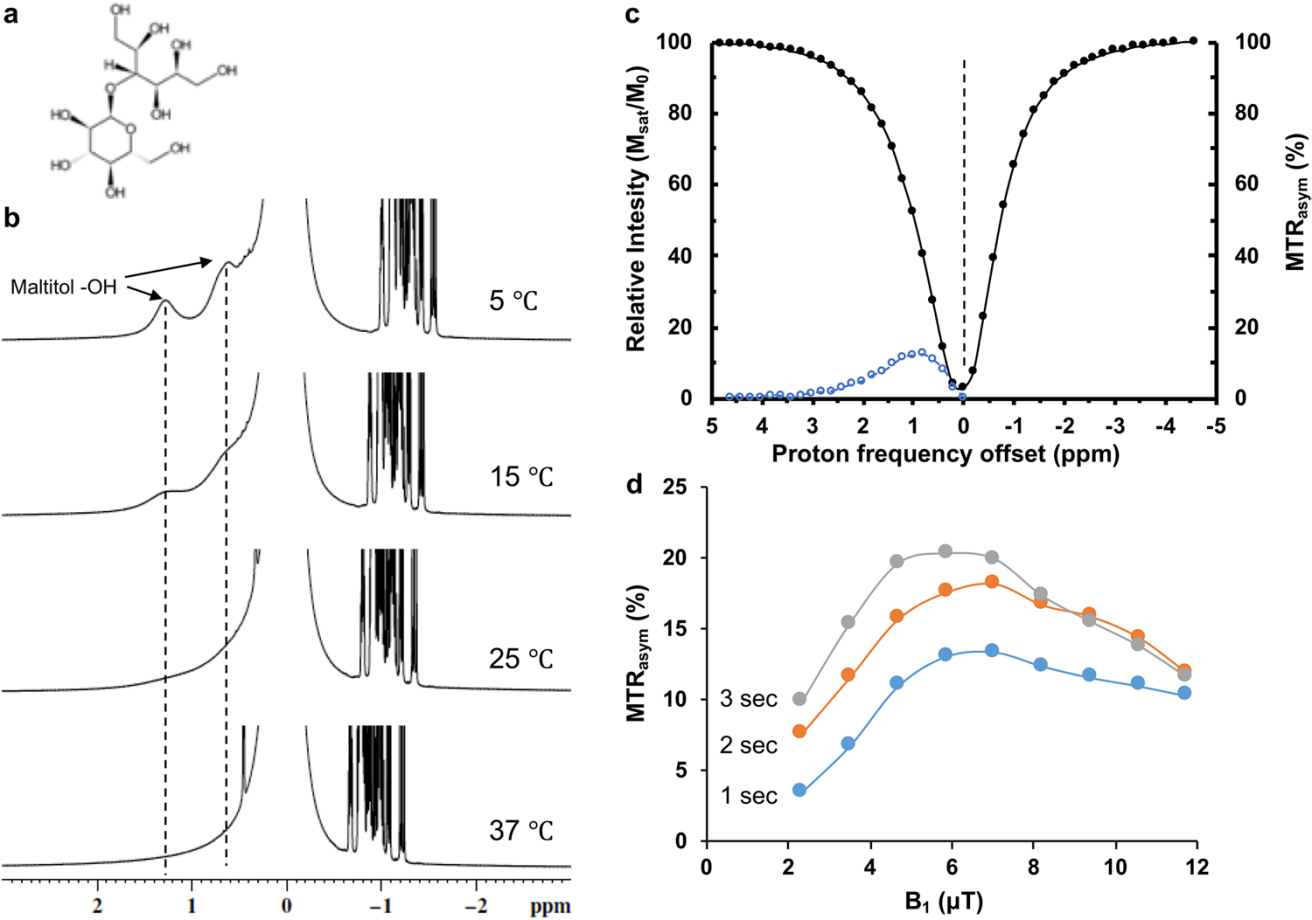
CEST effect from maltitol sweetener. (**a**) Chemical structure of maltitol. (**b**) High resolution NMR spectrum of 200 mM maltitol solution in PBS shows two peaks from exchangeable hydroxyl protons (-OH) respectively at 0.8 and 1.3 ppm at 5 °C. These peaks exchange broaden with increase in temperature and completely broadened at 37 °C. (**c**) Z-spectra (black) and asymmetry curves (blue) from 10 mM of maltitol show broad resonance (0–3 ppm) from exchangeable hydroxyl protons, which peaks at 1 ppm. (**d**) Saturation power and saturation dependence malCEST contrast. Higher B_1_ and saturation duration increase the malCEST effect.

Maltitol is a disaccharide of glucose and sorbitol, having 9 water exchangeable -OH groups (Fig. 1a). Chemical shift of the labile -OH protons of maltitol was determined using high-resolution nuclear magnetic resonance (NMR) spectroscopy. The spectra from 200 mM maltitol (pH 7) were acquired at different temperatures (5, 15, 25, & 37 °C) on a 400 MHz NMR spectrometer (Bruker, Germany). Two peaks at ~0.8 and ~1.3 ppm down field of water were detected due to slower exchange between maltitol -OH protons and bulk water at low temperatures (Fig. 1b). The -OH peaks were found to broaden at higher temperatures due to faster chemical exchange and completely disappear at 37 °C. The observation of -OH peaks at ~1 ppm downfield to water signal suggests the feasibility of CEST based experiments using maltitol.

### Evaluation of CEST effect from maltitol

The characterization of CEST effect from maltitol was performed by applying a continuous low power saturating RF pulses at frequencies gradually moving away from the reference water peak are visualized by plotting the water saturation as a function of saturation frequency. The z-spectra and z-spectral asymmetry (MTR_asym_) curves obtained from maltitol solution showed maximal malCEST effect at 1 ppm (Fig. 1c). The malCEST contrast from 10 mM maltitol solution *in vitro* was found to be maximum at ~6 µT saturation power (B_1_) for all saturation durations (Fig. 1d). For a given B_1_, higher malCEST contrast was observed with the increase in saturation duration (Fig. 1d).

### Concentration and pH dependence of malCEST contrast

**Figure 2:**
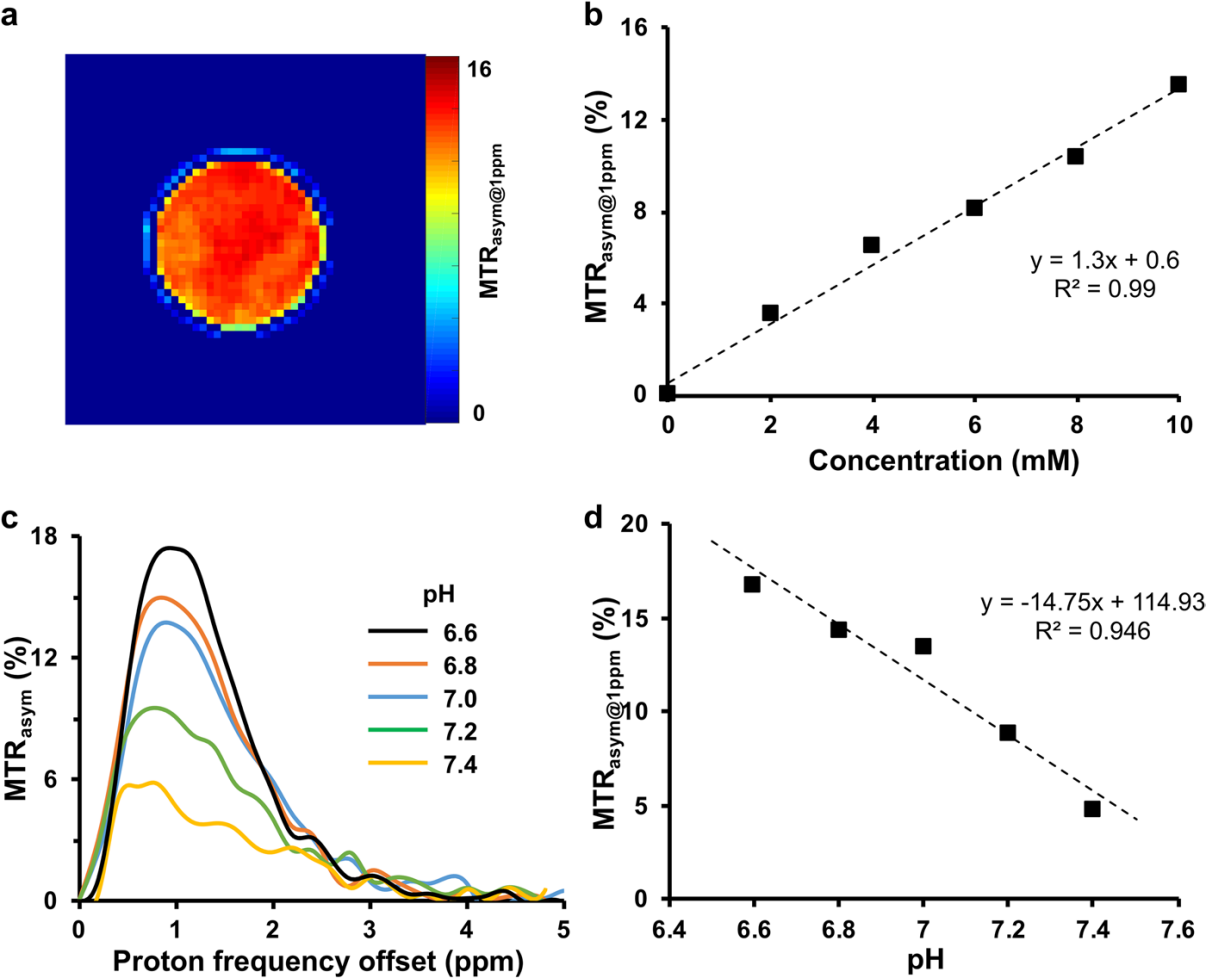
MalCEST map of maltitol. (**a**) malCEST map obtained from 10 mM maltitol solution phantom at 37 °C shows homogenous contrast. (**b**) Plot depicts the concentration dependent malCEST contrast. (**c**) Z-spectra asymmetry curves at different pH show higher malCEST contrast with decrease in pH. (**d**) inverse linear correlation between malCEST and pH was observed.

The concentration dependence of malCEST contrast was evaluated *in vitro* on solution phantoms prepared at physiological pH and temperature (37 °C). The MTR_asym@1ppm_ map from 10 mM phantom showed ~13% of malCEST contrast (Fig. 2a) and was linearly proportional to the maltitol concentration with a slope of 1.3% per mM of maltitol (Fig. 2b). The malCEST effect was found to be inversely proportional to pH (Fig. 2c,d). The data acquired from maltitol solution at varying pH depicted 14.8% increase in malCEST per unit decrease in pH. The malCEST map from 10 mM maltitol solution acquired on 7 T human MRI scanner is shown in the Supplementary Fig. 1.

### CEST MRI experiments in control rats

**Figure 3:**
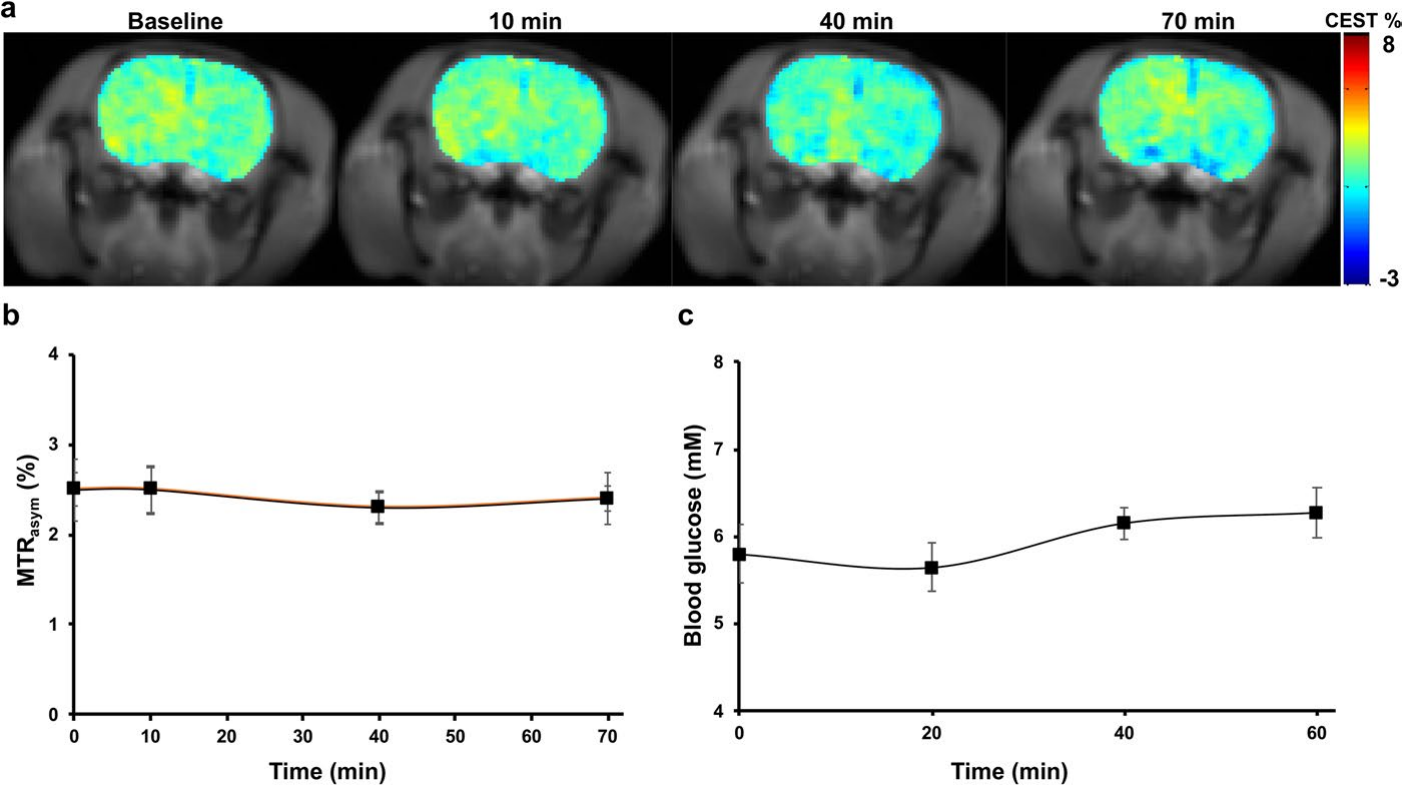
MalCEST imaging in a normal rat brain. (**a**) CEST MRI from a normal rat brain depicts no appreciable change in the malCEST contrast following injection of maltitol over 70 min. (**b**) Data from the normal rats (n = 3) shows no change in malCEST contrast in the brain during and following maltitol administration. (**c**) There was no observable change in the blood glucose levels post maltitol administration.

To assess whether maltitol crosses the intact BBB, an *in vivo* study was performed on control rats (n = 3). At the given saturation parameters (2.35 µT, 2 sec), a baseline 2–3% MTR_asym@1ppm_ was observed. This may be due to the endogenous metabolites with -OH groups present in the brain predominantly myo-inositol and glucose. The malCEST contrast maps from a normal rat brain were acquired at different time intervals over a period of 70 minutes during and following the intravenous injection of maltitol (Fig. 3a). No appreciable change in the malCEST contrast was observed in the normal rat brain over the period of 70 minutes (Fig. 3b) most likely due to the intact blood-brain barrier (BBB).

To evaluate the effect of intravenously injected maltitol on the blood glucose level, 50 µl of blood was collected every 20-minute time interval for up to period of 60 minutes. The blood glucose level during period of 60 minutes following the beginning of maltitol infusion was found to remain the same (Fig. 3c), indicating that there was no hydrolysis of maltitol into glucose and sorbitol. Further, the blood pH following maltitol administration was found to be unaltered until 60 minutes in three control rats.

### CEST experiments on glioma rats

**Figure 4:**
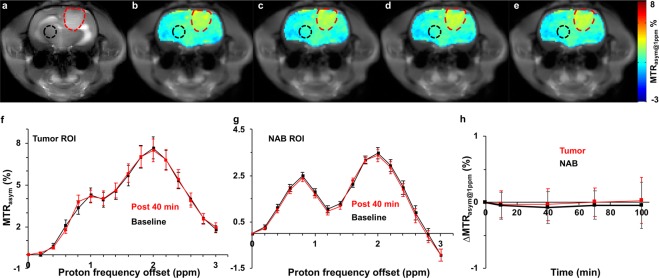
Normal saline perfused malCEST imaging in glioma. (**a**) Anatomical proton weighted image shows tumor as a hyperintense region. (**b**–**e**) MTR_asym@1ppm_ maps at different time points show no appreciable change in the CEST contrast at 1 ppm from tumor (red outline) and normal appearing brain (black outline) regions following administration of normal saline (**b** Pre-injection; **c**, 10 min; **d**, 40 min and **e**, 70 min post the beginning of normal saline administration). (**f**) MTR asymmetry curves from ROIs placed in the tumor region generated at baseline and 40 minutes post infusion of normal saline showing unaltered MTR_asym_. (**g**) Asymmetry curves from ROIs placed in the NAB region generated Pre- and Post 40 minutes showing no change in MTR_asym_ from normal saline. (**h**) ΔMTR_asym@1ppm_ (MTR_asym@1ppm_Post − MTR_asym@1ppm_Pre) contrast was unaltered during and following normal saline administration in both tumor and NAB ROIs.

**Figure 5:**
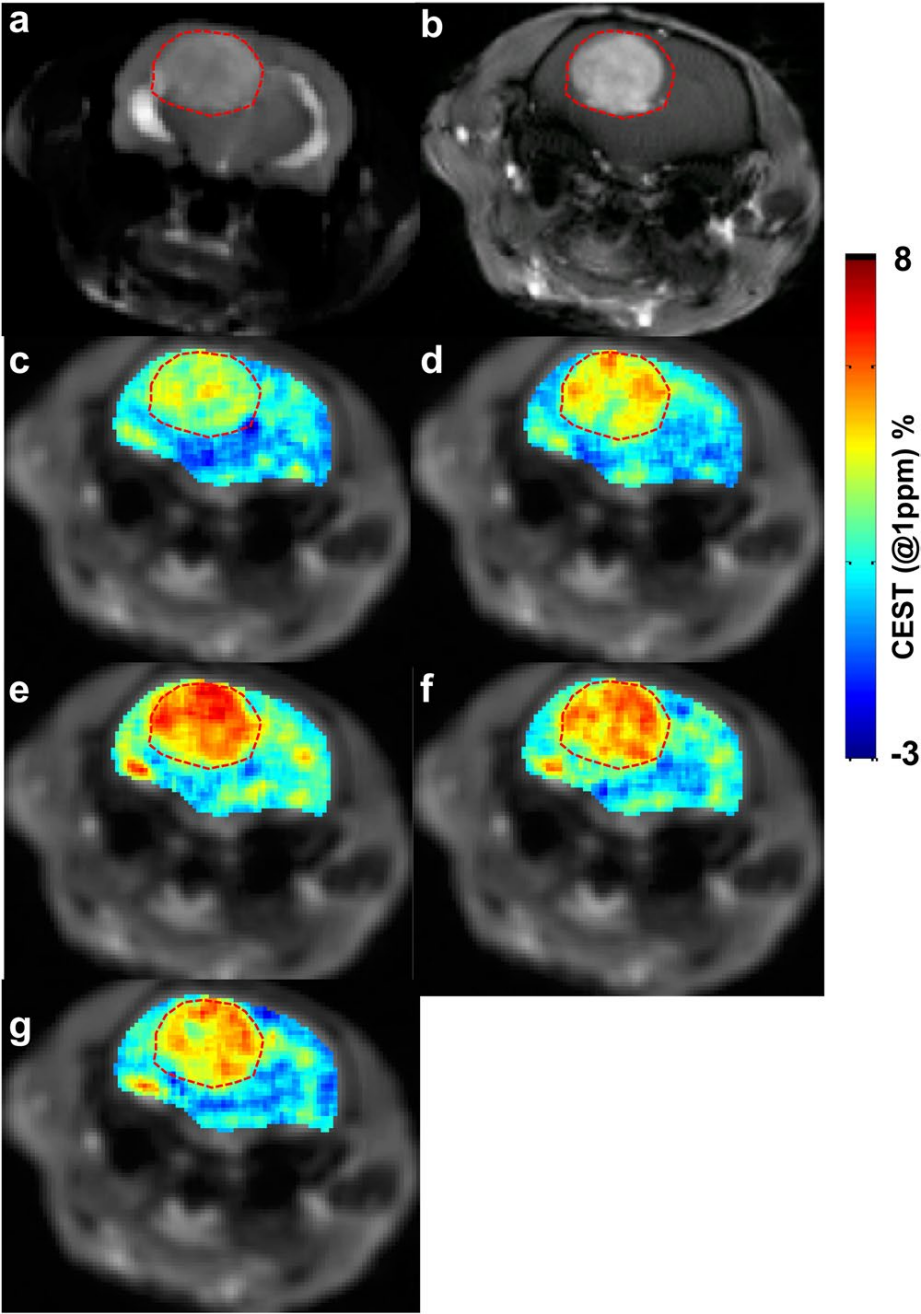
The malCEST map of a rat brain tumor. (**a**) Anatomical T2-weighted image from axial slice showing the location of tumor as hyper intense region in the rat brain. (**b**) The T1-weighted image post Gd-DTPA administration highlights the tumor areas in the brain. (**c**–**g**) malCEST map from rat brain tumor shows increased contrast in tumor region following intravenous injection of maltitol peaking at 40 minutes following the start of injection (**c**, Pre-injection; **d**, 10 min; **e**, 40 min; **f**, 70 min; and **g**, 100 minutes post the beginning of maltitol administration).

**Figure 6:**
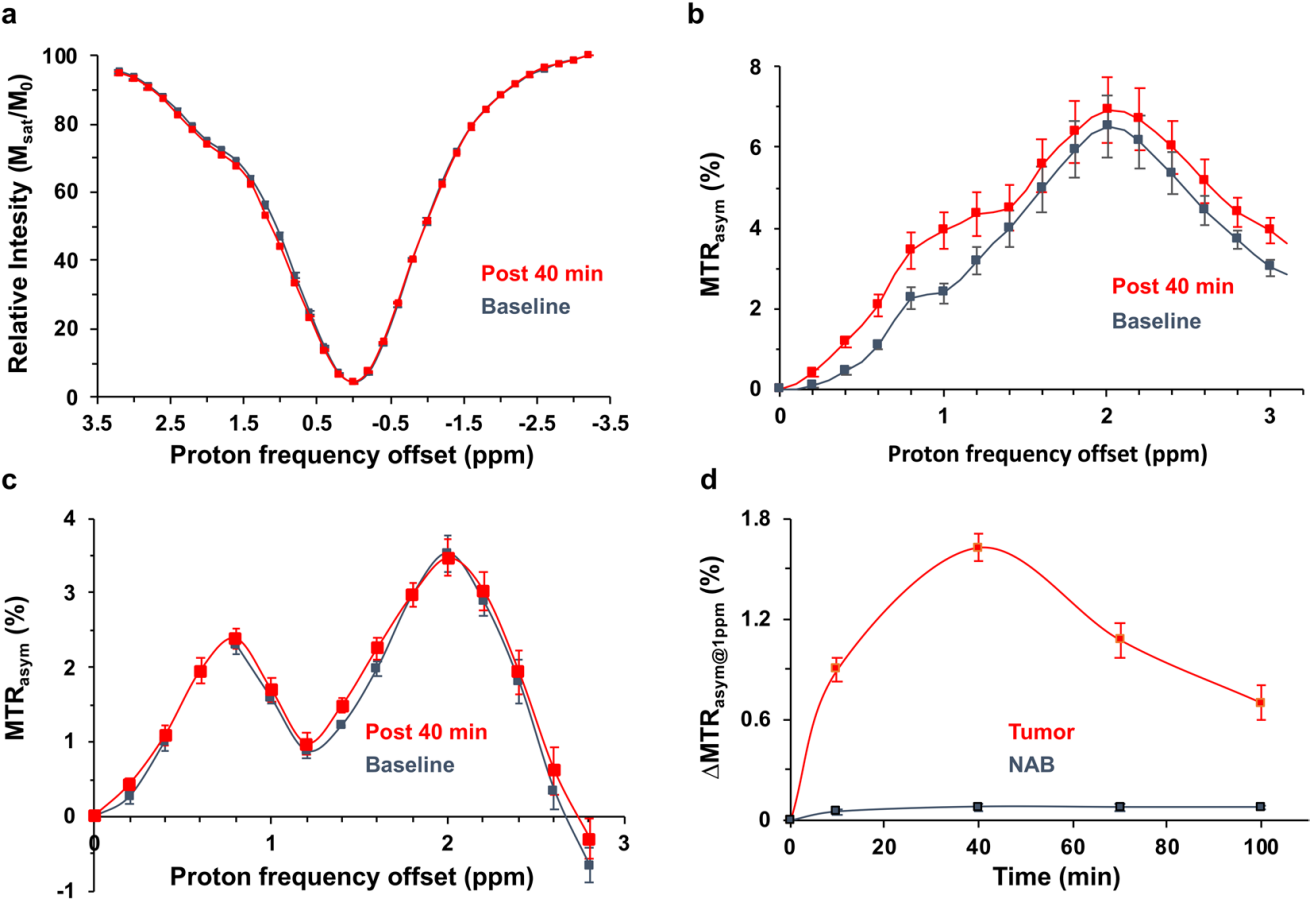
Z-spectra and MTR_asym_ analysis of tumor and normal appearing brain following maltitol administration. (**a**,**b**) Z-spectra and MTR asymmetry curves from ROIs placed in the tumor region generated at baseline and 40 minutes post infusion of maltitol show increased MTR_asym_ at 1 ppm (MTR_asym@1ppm_). (**c**) MTR_asym_ curves from ROIs placed in the normal appearing brain (NAB) region generated at baseline and post 40 minutes showing no change in MTR_asym_ due to intact BBB. (**d**) ΔMTR_asym@1ppm_ (MTR_asym@1ppm_Post − MTR_asym@1ppm_Pre) contrast at different time points peaks at 40 minutes in the tumor ROI while NAB was unaltered during and following maltitol administration.

To assess the potential of maltitol for providing CEST contrast to image cancer *in vivo*, we performed MR imaging experiments on rats carrying 9 L glioma. As the maltitol solution for MRI experiments was prepared in normal saline (NS), it is necessary to determine the effect of NS on the CEST contrast post intravenous administration. The MTR_asym_ maps at 1 ppm shown in Fig. 4 demonstrate no change in the CEST contrast pre and post-NS administration indicating NS does not contribute to the CEST changes in further experiments. Following this, we performed MR studies including T1/T2-weighted and CEST MRI in glioma carrying rats (n = 5) by administrating maltitol. The T2 weighted image showed hyperintensity in the tumor (Fig. 5a), correspondingly, Gd-DTPA enhanced T1-weighted image clearly highlighted the tumor region (Fig. 5b). The malCEST contrast measured post administration of maltitol highlighted only the tumor region (Fig. 5c–g) presumably due to the accumulation of maltitol in the extracellular and extravascular space (EES) because of the enhanced BBB permeability and retention effect. The MTR_asym_ curves obtained pre- and post-maltitol administration clearly showed increase in the CEST contrast at 1 ppm demonstrating that the change is due to EES accumulation of maltitol in the tumor (Pre: 2.33 ± 0.24%, Post 40 min: 3.95 ± 0.44%; p < 0.001) (Fig. 6a,b). There was no appreciable change in the malCEST contrast in the normal appearing brain regions (Pre: 1.62 ± 0.10%, Post 40 min: 1.69 ± 0.15%; p = 0.84) (Fig. 6c), which further confirms that maltitol does not cross the intact BBB. In the tumor, malCEST contrast peaked at ~40 minutes during the intravenous administration (Fig. 6d). The change in the malCEST contrast between tumor and contralateral normal appearing brain (NAB) ROIs post 40 minutes from the beginning of maltitol infusion was found to be statistically significant (Tumor: 1.62 ± 0.10%, NAB: 0.07 ± 0.02%; p < 0.001).

## Discussion

In this study, we showed the potential of a commonly used low calorific sweetener, maltitol, as an MRI contrast agent in imaging cancer by exploiting the exchange-based property of maltitol hydroxyl protons with those of bulk water. The chemical shift of the exchangeable hydroxyl protons of maltitol was found to be between 0.7–1.4 ppm downfield of water allowing its application as a possible CEST contrast agent. Being a small molecule, maltitol can easily accumulate in the cancerous tissues. Moreover, maltitol does not have a metabolic fate in the tumor due to the absence maltase enzyme responsible for the breakdown of maltitol into glucose and sorbitol.

D‐glucose and its analogues have shown potential as CEST contrast agents for the non-invasive detection of various cancers in preclinical and clinical studies^18,28–31,33,35–40^. Dynamic glucose-enhanced (glucoCEST) MRI has been used to study perfusion in cancer which corroborated with DCE MRI utilizing GBCAs. Recently, glucoCEST has been used to evaluate the efficacy of rapamycin, a glucose transporter blocker, in preclinical model of glioblastoma^20^. This study reported a higher glucoCEST contrast from tumor post-treatment with rapamycin. In addition, non-metabolized glucose analogues such as 3-O-methylglucose (3-OMG), have been exploited as CEST contrast agents for studies in cancer and stroke^24–26,28–30,32^. Further, another study has shown the use of a non-caloric sweetener, sucralose, as a CEST contrast agent to detect glioma in a preclinical model^41^. There are several other studies involving another glucose analogue, 2-deoxy-D-glucose (2DG) as CEST agent to image cerebral glucose uptake^27^ in cancer^35^ and Alzheimer’s Disease^42^.

In the normal brain, the malCEST contrast was unaltered following intravenous injection of maltitol suggesting that maltitol is unable to cross the BBB. However, in the brain tumor model with compromised BBB, the injected maltitol diffuses in the tumor EES and highlights the tumor areas. Significantly higher contrast was observed in the tumorous region at 40 minutes during maltitol infusion, while no change in the normal appearing brain region was observed. The spatial extent of malCEST enhancement appeared to broadly agree with that of the Gd-DTPA enhancement. The current study demonstrates the potential of an alternative high-resolution MR method to image cancers in preclinical models. The kinetics of malCEST contrast may be further evaluated in different cancer types and could potentially be used as a biomarker to differentiate cancer phenotypes.

Maltitol is FDA approved to be consumed orally at very high daily dose up to several grams. The toxicity profile of maltitol has been evaluated in human volunteers following varying oral dose administration, observed no maltitol related adverse effects except at higher doses (70 g/day) where 15–30% volunteers reported diarrhea^43^. Previous studies have shown no toxic effect of intravenously injected maltitol at a dose of ~1.25 g/kg in rats^44^. In the current study, we have used maltitol at a dose of 2.5 g/kg without any observable toxic effects. Since maltitol is not approved by the FDA for intravenous injection in humans, it may be a limiting factor for the clinical translation of this technique. We observed no noticeable side-effects in the rats at the dosage level used in this study. Further, consistent with a previous study, we observed no change in the blood glucose level post maltitol administration^44^. Oral vs intravenous administration has different fates of maltitol inside the body. Oral administration of maltitol has been shown to not increase the blood maltitol level due to its conversion into glucose and sorbitol by the action of maltase present in the gut^45^. Absence of maltase in the rest of the body may be the reason for the observed unaltered blood glucose levels after intravenous injection of maltitol.

Maltitol is reported to have very high clearance rate from the body as reported previously^44^. By 60 minutes following intravenous administration, maltitol is nearly cleared from the blood stream. This may be a reason for the lower retention of the maltitol in the EES resulting in lower observed changes in the MTR_assym@1ppm_. Also, it has been shown that intravenous injected maltitol is virtually cleared from the blood within an hour^44^. Further studies at different doses are needed to validate that the observed CEST contrast arises from maltitol accumulation in the tumor.

Though we have evaluated the concentration and pH dependence of the CEST effect from maltitol *in vitro*, this study lacks data on the differential dosing of malitol on the malCEST contrast *in vivo*. Further studies are required in tumor-bearing animals to optimize the dose for contrast enhancement from the EES accumulation of maltitol. Moreover, though the malCEST enhancement in glioma corroborated with the Gd-DTPA enhanced map, a direct comparison between the two techniques is beyond the scope of this study. It is noteworthy that CEST acquisition times are longer compared to GBCA based DCE MRI studies. Also, CEST MRI in the current study is limited to 2D acquisition of a thick slice to obtain sufficient signal-to-noise ratio, while the GBCA based studies can be performed at significantly higher in-plane resolution and 3D. Finally, the B_1_ power and duration optimization performed *in vitro* yielded higher optimal B_1_ (~6 μT) for observing malCEST, while a lower B_1_ (2.35 μT) was used for *in vivo* experiments. This was due to the reason that high saturation powers cause off-resonance saturation of water leading to the reduction in CEST amplitude, especially when the exchanging protons have a small chemical shift and slow chemical exchange rate^46–49^. Although, maltitol hydroxyl group exchange rate *in vitro* appears to be high, as the extracellular pH (pHe) in tumor tissue is lower than the normal tissue^50^, this would lead to a reduction in the exchange rate of the accumulated maltitol hydroxyl groups *in vivo*. Moreover, higher B_1_ power results lower SNR *in vivo* than *in vitro* as the water concentration is higher *in vitro* than *in vivo* (~70%)^51^.

In conclusion, this preliminary study paves the way for the development of maltitol and other maltitol derivatives as MRI contrast agents to detect cancer and monitor therapeutic response in preclinical studies.

## Materials and Methods

### Phantom preparation

All the solution phantoms were prepared in phosphate buffered saline (PBS) and the experiments were performed at 37 °C. For high-resolution ^1^H NMR spectroscopy, 200 mM of maltitol (Sigma Aldrich, USA) solution was prepared in PBS at pH 7. To measure the pH dependence of malCEST, phantoms with 10 mM maltitol concentration in PBS were prepared at a varying pH from 6.6 to 7.4 in step of 0.2 pH unit. The pH was adjusted using 1N NaOH/HCl. For measuring concentration dependence of malCEST contrast, phantoms with 2, 4, 6, 8, and 10 mM concentrations of maltitol were prepared in PBS at pH 7.

### Phantom imaging

High-resolution ^1^H NMR phantom experiments from 200 mM maltitol solution were performed on a vertical bore Bruker Avance DMX 400 MHz spectrometer (Bruker Corporation, Germany), equipped with a 5 mm PABBI proton probe using a single pulse experiment with parameters: TR = 4 s, number of averages = 128. ^1^H MR spectra were gathered at different temperatures (5, 15, 25, 37 °C). A narrow coaxial capillary containing D_2_O and TSP was used for locking and chemical shift reference, respectively.

Imaging was performed on a 9.4 T, 30 cm horizontal bore animal MRI scanner (Agilent, USA) interfaced to a Varian console, with a 20 mm volume coil (M2M Imaging, USA). A custom-written segmented spoiled GRE pulse sequence with a frequency selective continuous wave saturation preparation pulse was used to perform CEST experiments. The sequence parameters were: field of view (FOV) = 20×20 mm^2^, slice thickness = 10 mm, flip angle = 15°, TR = 6.2 ms, TE = 2.9 ms, matrix size = 128×128. For every 15 s, one saturation pulse was applied. CEST images were collected using variable saturation lengths (1 through 3 seconds) and saturation pulse amplitudes (B_1rms_: 2 to 12 µT). For concentration and pH dependent studies, CEST images were collected using 1 second saturation pulse at B_1rms_ of 7 µT for multiple frequencies (−3.4 to +3.4 ppm in 0.2 ppm steps) from bulk water. B_1_ and B_0_ field maps were also gathered and used to correct the CEST contrast maps. Briefly, CEST data, acquired in the neighborhood of ±1 ppm and WASSR maps were used to generate corrected malCEST images (±1 ppm) using a procedure similar to that described previously^41,52^.

Relative B_1_ maps were obtained using a magnetization prepared spoiled GRE method. For B_1_ correction, two images were obtained using preparation square pulses with duration (τ) and flip angles of 30° and 60°. The RF pulse amplitude for a 30° flip angle was used as the reference B_1_ or B_1ref_. Flip angle (θ) maps were generated by solving the equation:

$$\frac{\cos (2\varphi )}{\cos (\varphi )}=\frac{S(2\varphi )}{S(\varphi )}$$

where S(ø) and S(2ø) denote pixel signals in an image with preparation flip angle ø and 2ø respectively. From the flip angle map, a B_1_ field map can be obtained using the relation, B_1_ = ø*(360τ)^−1^. The coefficient B_1_/B_1ref_ was used for B_1_ correction of the malCEST contrast at 1 ppm.

### Rat tumor model preparation

The Institutional Animal Care and Use Committees (IACUC) of the University of Pennsylvania approved experimental protocols, and all experiments were carried out in accordance with approved IACUC guidelines.

To validate the malCEST *in vivo*, a rat brain tumor model was used. To develop intracranial tumors, 9 L gliosarcoma cells were used. Syngeneic female Fisher rats (F344/NCR, four-six weeks old) weighing 130–150 grams were used to generate tumor-bearing rats as described previously^41,53^. General anesthesia was induced using 2% isoflurane mixed with 1 liter/min oxygen. A 10 µl suspension of 50‚000 9L cells in phosphate buffered saline was injected into the cortex at a depth of 3 mm with a Hamilton syringe and a 30-gauge needle using stereotactic apparatus (3 mm lateral and 3 mm posterior to the bregma). Five weeks after implantation of tumor cells rats were subjected to MRI.

### Rat MR imaging

A total of 11 rats were used for imaging. Eight rats were administered with 9 L glioma cells for CEST studies following normal saline (NS, n = 3) and maltitol administration (n = 5). While the remaining 3 rats were used as healthy controls for CEST studies following maltitol administration.

Rats were anesthetized with isoflurane (3% for induction, 1.5% maintenance) and a polyethylene catheter (PE50) was inserted into the tail vein for maltitol or NS injection. MRI imaging was performed on tumor bearing rats five weeks after 9 L cell implantation. Rats were transferred to a 9.4 T horizontal bore small animal MR scanner (Varian, Palo Alto, CA) and placed in a 35 mm diameter commercial quadrature proton coil (m2m Imaging Corp., Cleveland, OH). Animals were kept under anesthesia (1.5% isoflurane in 1 liter/min oxygen) and their body temperature maintained with the air generated and blowing through a heater (SA Instruments, Inc., Stony Brook, NY). Respiration and body temperature were continuously monitored using an MRI compatible small animal monitoring system (SA Instruments, Inc., Stony Brook, NY).

CEST imaging of normal rat brain and rat brain tumors was performed using a similar pulse sequence and parameters as described in the case of phantom imaging except a FOV = 35 × 35 mm^2^, slice thickness = 3 mm, matrix size = 128×128, B_1_ = 2.35 µT, saturation length = 2 s and TR = 8 s. CEST images were collected at multiple frequencies (−3.4 to + 3.4 ppm in 0.2 ppm steps) from bulk water. After baseline imaging the rats were injected maltitol solution at bolus variable rate^54^ through the catheter inserted in a tail vein for a period of 60 minutes. Animals received a 300 µl bolus of 1 M maltitol (per 200 gm body weight) intravenously. The rate of the infusion was decreased manually every 1 min followed a decreasing exponential function during the first 8 min and was constant for the remainder of the experiment. The total volume infused was ~1.5 ml. CEST acquisitions were started immediately with the beginning of infusion and acquired at 30-min time interval for a total period of 120 minutes. After CEST imaging, gadolinium-diethylenetriamine pentaacetic acid (Gd-DTPA) (0.3 mmol/kg) was injected through tail vein and T1-weighted imaging was performed on the same anatomical slice used for CEST with GRE (gradient echo) sequence using following parameters: FOV = 35×35 mm^2^, Flip Angle = 8°, Slice thickness = 3 mm, matrix size = 128×128, TR = 6.2 ms, TE = 2.8 ms, and averages = 12.

Similar CEST imaging protocol was used following infusion of NS in glioma bearing rats. Blood glucose and pH measurement at 20, 40 and 60 minutes was also performed using a blood gas analyzer following the administration of maltitol in normal rats.

### Image processing

For each pixel in the CEST-weighted images, the z-spectrum was fit to a quadratic polynomial and shifted by the corresponding B_0_ offset. B_0_ corrected CEST-weighted images are obtained by evaluating the fit at ±1 ppm (S_+1ppm_ and S_−1ppm_, respectively), and CEST contrast is given by the MTR asymmetry evaluated by Eq. (1).

1 $$MT{R}_{asym}( \% )=100\times (\frac{{S}_{-ve}-{S}_{+ve}}{{S}_{0}})$$

where *S*_0_ is image with 20 ppm offset saturation. B_1_ inhomogeneity was then corrected linearly by pixel-wise dividing by the relative B_1_ map. ROIs were manually drawn on tumor and normal appearing brain regions. All image processing and data analysis were performed using software routines written in MATLAB (R2015b) as described in detail elsewhere^14,55^.

### Statistics

Statistical analyses were performed using SPSS. A student’s t-test was performed to compare the changes in the CEST contrast in the normal appearing brain and tumor ROIs following the administration of maltitol intravenously. The data is presented as mean ± standard error.

## Supplementary information

Supplementary Figure 1
