# Supplementary material for: Sugar alcohol provides imaging contrast in cancer detection
Source: Sci Rep. 2019 Jul 31;9:11092. doi: 10.1038/s41598-019-47275-5 (PMC6668433; doi:10.1038/s41598-019-47275-5)

Sugar alcohol provides imaging contrast in cancer detection

Puneet Bagga[1], Neil Wilson[1], Laurie Rich[1], Francesco M. Marincola[2], Mitchell D. Schnall[1], Hari Hariharan[1], Mohammad Haris[1,2,3], Ravinder Reddy[1]

[1] Center for Magnetic Resonance and Optical Imaging, University of Pennsylvania, Philadelphia, Pennsylvania, USA
[2] Research Branch, Sidra Medical and Research Center, Doha, Qatar
[3] Laboratory Animal Research Center, Qatar University, Doha, Qatar

Supplementary Figure 1. MalCEST effect from 10mM maltitol phantom at 7T human scanner. The CEST imaging was performed using 3 μT B_1 and 2 sec saturation duration. Around 11% contrast was observed.

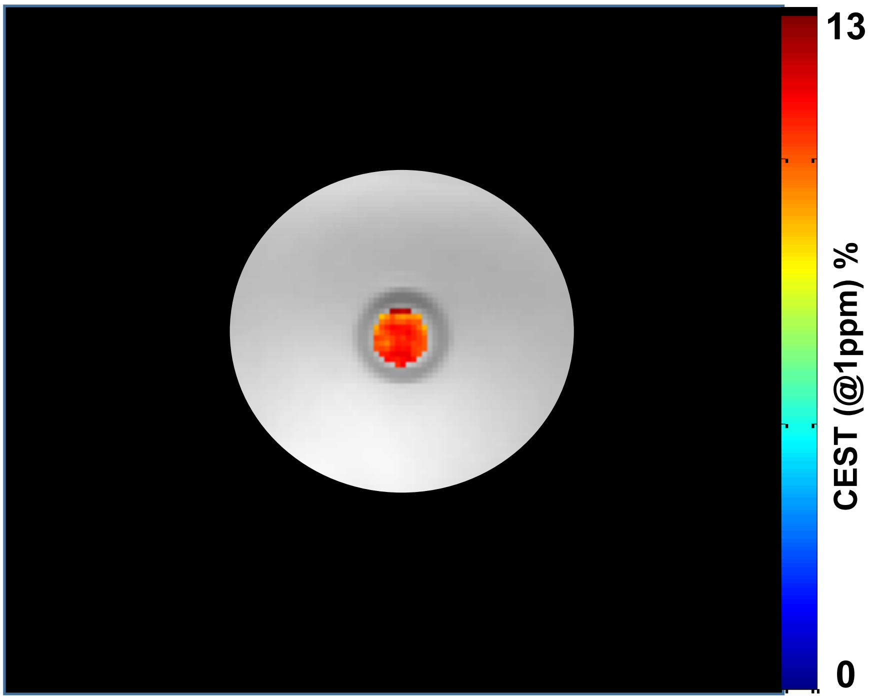

Supplement: Supplementary file 1 — Supplementary Figure 1 [file 41598_2019_47275_MOESM1_ESM.pdf]
